# Real‐World Data: An Unrealized Opportunity in Global Health?

**DOI:** 10.1002/cpt.1476

**Published:** 2019-06-12

**Authors:** Jeffrey S. Barrett, Penny M. Heaton

**Affiliations:** ^1^ Bill & Melinda Gates Medical Research Institute Cambridge Massachusetts USA

The positive perspective for real‐world data (RWD) and early evidence of improved decision making is largely realized by development strategies focused on the developed world. Although the use of RWD to bridge populations for safety and efficacy works well in some instances, this bridging exercise is often not appropriate in a global health context. Efforts to include RWD into research and development (R&D) strategies are ongoing for low‐income countries with great expectation to inform translational medicine paradigms for these populations.

The benefit of RWD to inform various aspects of drug development is well supported^1^, ^2^, ^3^ with great expectation for expanded utilization.^4^, ^5^ The positive perspective for RWD and early evidence of improved decision making is largely realized by development strategies focused for the developed world (i.e., advanced economies with advanced technological infrastructure or high‐income countries (HICs)). Much of what we consider the modern era in drug development has occurred over the past 100 years (see **Figure** **1**, bottom panel). The history of drug development and the pharmaceutical industry is very much associated with the necessity of manufacturing and distributing adequate quantities of drug products to HICs. Coincidentally, regulation of the processes underlying the R&D and manufacturing became a necessity often in response to tragedy (e.g., thalidomide in pregnant women in the 1950s) with an eventual global regulatory oversight in place for the developed world. Ironically, many of these innovations and safety‐nets added to the drug development evolution were born out of evidence generated by RWD.

**Figure 1:**
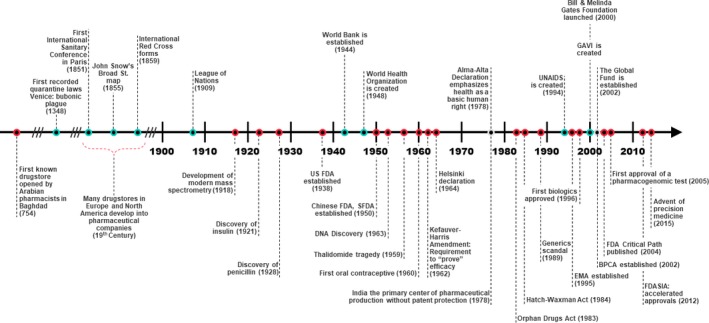
Timeline of events defining the pharmaceutical industry in the modern era (bottom panel) in relation to major global health milestones (top panel). BPCA, Best Pharmaceuticals for Children Act; EMA, European Medicines Agency; FDA, US Food and Drug Administration; GAVI, Vaccine Alliance; SFDA, China Food and Drug Administration; SIA, Safety and Innovation Act; UNAIDS, Joint United Nations Programme on HIV and AIDS.

The path for RWD utilization in the global health space (low/middle income countries) is not straightforward and additional challenges exist. Although the use of RWD to bridge populations for safety and efficacy works well in some instances within the developed world, this bridging exercise is often not appropriate in a more global context. Reasons for this can be due to a variety of factors, including differences in the standards of care, heterogeneous populations, societal structure/network, migration, and adherence. Some of these issues could be addressed by increasing the availability and utilization of RWD in the different regions of the world; however, the assumption that such data already exist or are accessible is often invalid. The trajectory of product development in and for developing or low‐income countries (LICs) has been very different than in the United States and other developed countries. Historically, products have been developed for the affluent world and then used in LICs with little or no data in those populations. This has changed over the last few decades. The Rotavirus vaccine is one of the first examples with early recognition that studies in LICs were needed to evaluate safety and establish efficacy of this oral vaccine, given that other oral vaccines (e.g., oral poliovirus vaccine) have lower efficacy in those populations. In fact, this is an example when RWD on polio/cholera vaccines contributed to decision making and study design for clinical trials in the developing world. In general, however, global health development timelines lag often due to unclear factors driving the understanding of disease epidemiology and progression and the lack of data documenting the global burden of disease (see **Figure** **1**, top panel). Complicating the global health trajectory is the lack of infrastructure to support well‐controlled clinical trials and the local regulatory environment to review and provide guidance to sponsors’ development plans.

Furthermore, much of the difference in the RWD availability between HICs and LICs lies in the infrastructure to support routine clinical care and the economics of the respective healthcare systems used to support their populations. If one considers the most common forms of RWD to include electronic medical records, electronic health records, claims databases, health surveys, patient registries, data from health‐related applications and mobile devices, and data from social media, it is easy to conclude that most of these data sources simply do not exist in LICs. With respect to actual RWD generated in global health target populations, this is accomplished with limited capacity to date given the difficulty gathering the data for the most part. The expectation is that such RWD will increase dramatically as LICs’ economies improve and governments have greater focus on healthcare and healthcare costs. Although not always optimal, most countries have some form of Civil Registration and Vital Statistics systems to record births, deaths, and causes of death. As these improve, they may represent another source of RWD that could be utilized for product development.

There are good examples, however, of early efforts to generate and exploit RWD to estimate the global burden of disease and map the epidemiology within geographic regions to identify the target population of interest and ensure that study enrollment is matched to areas where the global burden of disease is greatest.^6^ Ongoing efforts also seek to explore the generalizability of studies in these regions across other geographic areas. The validation of factors that predict disease across regions is one goal of this work that would have implications in the design of future trials and policy discussions and decisions regarding actual implementation at the patient level. Of course, the future availability of longitudinal RWD for this purpose would be of great value. **Table** **1** provides a representative, although not exhaustive, snapshot of current RWD sources in LICs. The sources invested in the generation of these data are uniquely focused in global health matters and the various Centers for Disease Control around the world, universities with longstanding research in global health, and the countries in which the patient populations reside have formed alliances to generate these data with high fidelity. Other organizations, such as the Institute for Health Metric Evaluation (IHME), collaborate extensively with the World Health Organization to ensure that the survey‐based data that inform the global burden of disease estimates^7^ are both accurate and accessible.

**Table 1:** Examples of RWD that would inform global health research and development for infectious disease

Data type	Sources and current availability
Global Burden of Disease	WHO, IHME^a^ (Lancet publications and on‐line); Academic centers of excellence through country‐specific alliances (e.g., LSHTM)
DALY estimates	IHME (GBD project)
Population risk factors	IHME, GHDx; CDC and equivalent country‐specific organizations
Force of infection	Clinical trial data (disease networks, publications); Academic centers (e.g., Imperial College, Institute Pasteur); IDM
Longitudinal patient‐level data	Clinical trial data (CPTR, TB, and malaria networks); EHR data (India only); Natural History Data (CDC and equivalents, foundations^a^)

CDC, Centers for Disease Control; CPTR, Critical Path to TB Drug Regimens; DALY, disability‐adjusted life year; EHR, electronic health record; GBD, global burden of disease; GHDx, Global Health Data Exchange; IDM, Institute for Disease Modeling; IHME, Institute for Health Metric Evaluation; LSHTM, London School of Hygiene and Tropical Medicine; RWD, real‐world data; TB, tuberculosis; WHO, World Health Organization.

^a^For example, KNCV Tuberculosis Foundation, The Hague, The Netherlands.

Singh *et al*.^4^ have recently reviewed the status and potential impact of RWD on modern drug R&D. Their perspective is uniquely focused on the developed world, consistent with the marketplace incentive, of course, and is strongly endorsed by regulatory authorities (US Food and Drug Administration guidance). The authors recommend that pharma needs to invest in making better use of electronic health records and their linkage to molecular databases (within the right governance and technology frameworks) to accelerate the generation of real‐world evidence relevant to clinical research and drug development. The authors identify the need for more precompetitive collaboration to grow this capability, especially given the demands for precision medicine research. They further suggest richer academic–industry–government partnerships assuming the willingness of governments to provide industry with access to anonymized health data and working collaboratively across academic centers. Finally, the authors envision these opportunities to scale up will also help to stimulate improvements in the data quality and interoperability of RWD sources across healthcare and academia. Hence, the benefit of RWD for product development in HICs is starting to be realized. These benefits should be considered for LICs as well. RWD has the potential to streamline product development, appropriately bridge data across populations, and ensure safety and efficacy in LIC populations and the desired public health impact.

There is currently no organization that tracks RWD sources in LICs that would serve as a hub for researchers internationally. Support from the Gates Foundation has indeed focused the efforts of groups, such as the IHME^8^ and the Institute for Disease Modeling,^9^ to collect some RWD of specific interest, as alluded to previously, but increased interest within these LICs represents the greatest source of optimism around the evolution of such data. China specifically has made substantial investment in the policy and infrastructure needed to capture RWD in a more consistent manner as well as support the creation of disease registries that would inform therapy in high prevalence diseases.^10^ There is also recognition in China that this progress must be further supported by strategies that improve the quality and usefulness of such data.

Access to RWD is complicated by the issues of ownership, deidentification, and privacy, all of which are changing dynamically in response to legal, political, and scientific motivations, which seldom act in a synergistic manner. It would be nice to assume that data stakeholders would adhere to open science principles, but this cannot be guaranteed at this stage. Thus, consumers of these data must negotiate with individual countries and/or institutions now with the typical nondisclosure agreement, data sharing or transfer agreement, and an agreement that articulates the extent to which individual sources can be presented, filed (regulatorily), or otherwise shared in any manner. The benefit of incentivizing the process will only be realized once a market for these data is created or through the philanthropy of the global health ecosystem. What is clear is that the best and most informative RWD to facilitate global health R&D is yet to be generated and will most likely include detailed the biomarker, genomic, and relevant clinical end points superior to what has been generated in the past.

Much of the promise of RWD and real‐world evidence is the mechanism to provide supportive and complimentary evidence to the randomized controlled trial especially in clinical settings and/or populations that are difficult to study. The necessity to be both rigorous and quantitative obligates a data‐centric perspective to this endeavor and an understanding of both data and knowledge gaps. Beyond understanding is the recognition that we fill the most influential gaps via targeted investigation and that we leverage the exiting data, models, and knowledge to conduct informative trials with a high probability of success. Although there are well‐known limitations to the current implementation of RWD to inform decisions for R&D targeted to global health, there is strong commitment to challenge conventional paradigms and deliver impact in lives saved where the global burden of disease is greatest. Challenges exist but opportunity is already there and more is emerging. Global health product developers take note.

## Funding

The Bill & Melinda Gates Medical Research Institute is a wholly owned subsidiary of the Bill & Melinda Gates Medical Research Foundation. No additional sources of funding were provided to support this research.

## Conflict of Interest

The authors declared no competing interests for this work.
